# The Book World of Medicine and Science

**Published:** 1894-05-05

**Authors:** 


### The Book World of Medicine and Science.

Infectious Diseases: Notification and Prevention. By Louis C. Parkes, M.D., D.P.H. (Published by H. K. Lewis. Price 4s. 6d.)

Dr. Parkes has supplied both the medical officer of health and the general practitioner with a most useful handbook, and of such convenient size that it can always be carried in the pocket. In the small space of about 70 pages he gives the sections relating to infectious diseases contained in the Notification and Prevention Acts of 1889 and 1890, and the two Public Health Acts, with explanatory notes after each section, whereby a wearisome search through the different Acts is obviated; attention is also called to the points in which the later Public Health Act differs from that of 1875. In some few instances the author indulges in criticisms on the provisions of the law which appear somewhat out of place in a book of reference, especially as the law as laid down has to be carried out whether an individual here and there entirely approves of its provisions or not. Further useful information follows regarding the removal of persons suffering from infectious diseases, also the complete regulation of the Metropolitan Asylums Board Ambulance Service, with directions when and where to apply, also a list of all the fever hospitals under the same Board, with their regulations and admission fees, and finally the local Government Board orders regarding special epidemic diseases, notably cholera.

The second part of the volume contains a list of infectious diseases, with incubation, quarantine, and infective periods of each, the sources of infection, and a table of the usual diagnostic signs, all excellently arranged and printed clearly for easy reference. A most useful part of this volume treats of "Infectious Outbreaks in Schools." Here Dr. Parkes discusses very clearly and fairly the advisability or otherwise of breaking up a school when an outbreak of infectious disease. In coming to a decision much must of course depend on the accommodation that exists for complete isolation, and the nature of the outbreak. Thus Dr. Parkes considers careful isolation sufficient when the outbreak is one of measles, German measles, mumps, chicken-pox, or influenza; but if it be scarlet fever it is frequently wiser to disperse the school, merely keeping those who are attacked by the disease, and either nursing them in the sanatorium if suitable, or sending them to a fever hospital. The reason why this exception should be made in the case of scarlet fever is that the disease often takes a severe form where a number of persons are together, especially if ventilation is not very carefully regulated, and also the after results of scarlet fever are often serious, so that an epidemic of this disease is very serious. Dr. Parkes gives school authorities excellent advice when he counsels them "not to remedy any drainage defects during term time, since the opening up of the ground around defective or obstructed drains, and the removal of defective internal house pipes and fittings is far more likely to aggravate disaster than to have any beneficial effect."

Another excellent piece of advice is that where vaccination or re-vaccination is necessary when large numbers are congregated together only a few should be done at once and at intervals which will ensure greater care in dressing inflamed arms. This advice might well be followed not only in schools, but in hospitals where it is too frequently the custom to vaccinate at the same time large numbers of the nursing staff.

The directions for preserving milk pure and for filtering water, ventilating dormitories and class-rooms for isolation at home, and disinfection after infectious disease are most complete and admirable. It is customary to advise removal of carpets in case of infectious disease under all circumstances, but we are glad to see common-sense warnings as the following given that when the boards are of a porous nature and there are cracks between it is far better to have the carpet up and send it to be disinfected by steam at the end of the illness. Also that when clothes are disinfected those worn by the patient immediately before the beginning of his illness should also be thoroughly disinfected.

This excellent little handbook concludes with the Local Government Board's directions regarding the relations of the medical officer of health and the medical practitioners of his district, directions which will assuredly solve many doubtful points regarding etiquette.

Although primarily written for medical men, this volume will be found very useful for nurses, and we would specially recommend it to district nurses.

The Students' Introductory Handbook of Systematic Botany. By Joseph W. Oliver. (London: Blackie and Son, Limited. Crown cloth, 4s. 6d.)

In these days we suffer from a plethora of text-books which profess to render the path of the aspiring student of science, or the anxious examinee (not necessarily "convertible terms") easier to traverse than heretofore. But one is sometimes compelled to regret that those persons who undertake the responsibilities which the authorship of a text-book involves, should sometimes neglect to keep pace with the current advance in the particular science which they profess to expound. It is due to such carelessness that antiquated, or even erroneous notions are served up before the unsuspecting student, who thenceforth proceeds to place himself in the uncomfortable position of a certain foreign nobleman, who after having, with the expenditure of considerable energy, acquired fluency in speaking English, visited this country, only to find that he had thoroughly mastered a broad Lancashire dialect. We confess to a feeling of regret in turning over the pages of the book before us, for although the part which deals with the flowering plant is fairly well done, the earlier and equally important sections dealing with the cryptogams betray complete lack of appreciation of the work which has been accomplished in this department of botany within recent years. For example, in the vascular cryptogams it has become clearly recognised that heterospory is a phenomenon which has appeared independently in several groups of this most important series of plants, and with the perception of this fact, our insight into the mutual affinities exhibited by the members of the class has been immensely advanced. And yet we find, in a text-book dated 1894, all this advance entirely neglected, and old exploded views confidently put forward. Again, in the section on the Muscineæ, the Riccias are stated to be aquatic plants, which is, however, not true either of the greater part, or of the more characteristic species, of the genus. In the Gymnosperms also, though the author hints in a footnote that the statements contained in the text respecting the germination of the pollen have been rendered doubtful, he might with advantage have gone further and given what is the true account, and which has now been common knowledge for some time, owing to the researches of Belajeff and Strasburger. The same remarks will apply, *mutatis mutandis*, to the statements on p. 163 respecting the pollen grains of angiosperms. On the whole the part treating of flowering plants is perhaps the best part of the book, but here again one meets with evidence which seems to point to an ignorance of the best German work. For example, the older view of the morphology of the Fumitory flower is given instead of Eichler's explanation, which is almost certainly the correct one. Altogether one misses throughout the book a really critical knowledge of the subject, although certain parts of it are well worth reading, as giving in a condensed form a summary of the economic uses of plants, a department often somewhat unceremoniously dismissed by professed botanists as being really foreign to their science.

